# Embedded upper end of double J stent at the site of renal pelvis injury following percutaneous nephrolithotomy: a rare complication

**DOI:** 10.1186/s12894-020-00673-8

**Published:** 2020-07-17

**Authors:** Fayez T. Hammad

**Affiliations:** Department of Surgery, College of Medicine and Health Sciences, United Arab Emirates University and Mediclinic Al Ain, Po Box 17666, Al Ain, UAE

**Keywords:** Embedded double J stent, Percutaneous nephrolithotomy, Injury, Case report

## Abstract

**Background:**

Injury of the renal collecting system is a well-known complication of percutaneous nephrolithotomy (PNL). Large injuries may cause excessive bleeding and fluid extravasation and require adequate drainage using several modalities such placement of JJ stents. Herein, we report on two cases in which the upper coil of the JJ stent got buried in the fibrous tissues which formed due to an injury of the collecting system during PNL.

**Case presentation:**

40 years old male and 32 years old female underwent standard PNL for partial and total staghorn calculi, respectively. During the procedure in both cases, the renal pelvis was injured. In both cases, JJ stent was used to drain the collecting system. Trial to remove the JJ stent 6 weeks following the procedure failed because the upper coils of the stents were embedded in the fibrous tissues at the perforation site. Laser incision of the fibrous tissues and releasing the upper coil of the stents were performed using percutaneous approach in the first case and flexible ureterorenoscopy (fURS) in the second patient. The procedures were uneventful in both cases.

**Conclusion:**

This is the first report of embedded JJ stents which got buried by fibrous tissues at the site of collecting system injury that occurred during PNL. To prevent this complication in such cases, we suggest draining the collecting system using nephrostomy tube instead of JJ stent. Alternatively, the upper coil of the stent should be placed away from the injury site.

## Background

Injury and perforation of the renal collecting system is a well-documented complication of percutaneous nephrolithotomy (PNL) [1–3]. Major injuries usually result in excessive bleeding or fluid extravasation and may necessitate cessation of the procedure and draining of the collecting system using nephrostomy tube, peri-renal drain, JJ stent or a combination of these procedures [2]. Herein, we report on two cases in which the upper coil of the JJ stent got embedded in the fibrous tissues which formed at the site of the collecting system injury.

## Case presentation

### Case-1

**Fig. 1:**
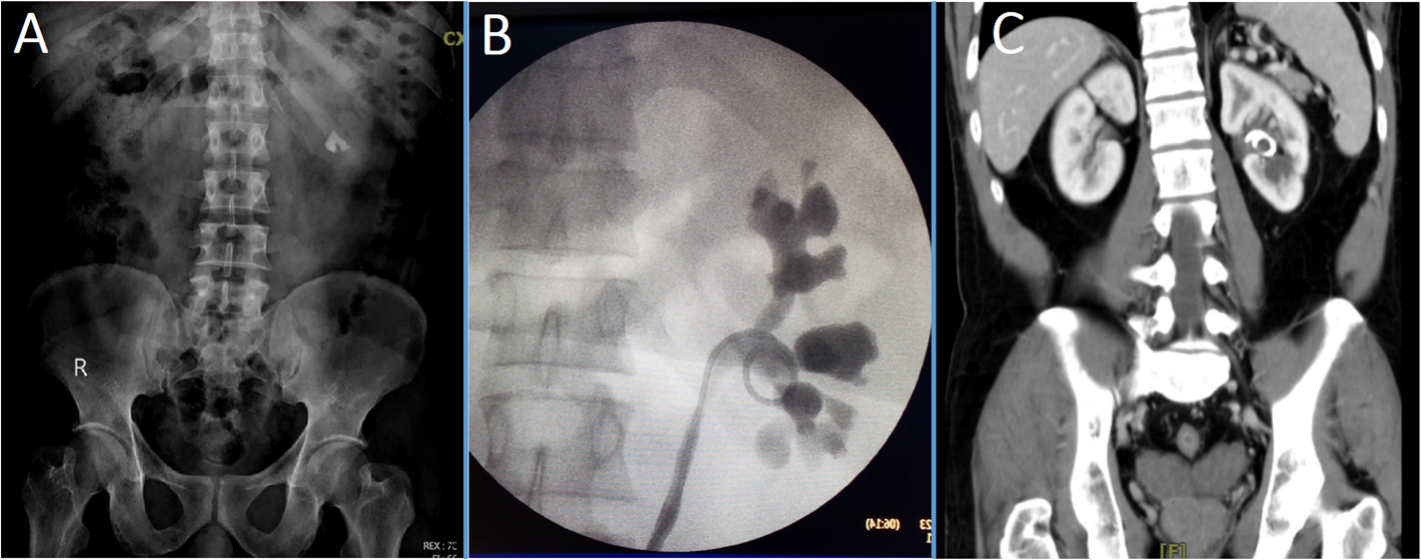
Imaging of the first case. **a**, Pre-operative plain x-ray with partial staghorn calculus; **b**, Retrograde pyelography showing the loop of the JJ stent outside the collecting system and the narrow renal pelvis; **c**, CT scan showing the upper coil of the JJ stent

A 40 years old male presented for the first time with left renal pain. Imaging showed left partial staghorn stone (Fig. 1a). He underwent single port, lower pole, prone PNL using Amplatz dilators. A combination of ultrasonic and pneumatic modality were used to fragment the stone. Towards the end of the procedure, and after complete stone clearance, we have noticed a perforation in the renal pelvis caused by the tip of the 30 Fr sheath. As there was minimal bleeding, the collecting system was drained only by antegrade placement of a JJ stent. Six weeks later, a trial to remove the JJ stent failed because the upper coil of the stent was found to be embedded in fibrous tissues at the site of the previous perforation as shown by the retrograde pyelography (Fig. 1b). A trial of flexible ureterorenoscopy (fURS) and laser incision of the fibrous tissues encasing the stent was not possible, so this was performed by percutaneous approach at a later date. The procedure was uneventful with minimal bleeding.

### Case-2

**Fig. 2:**
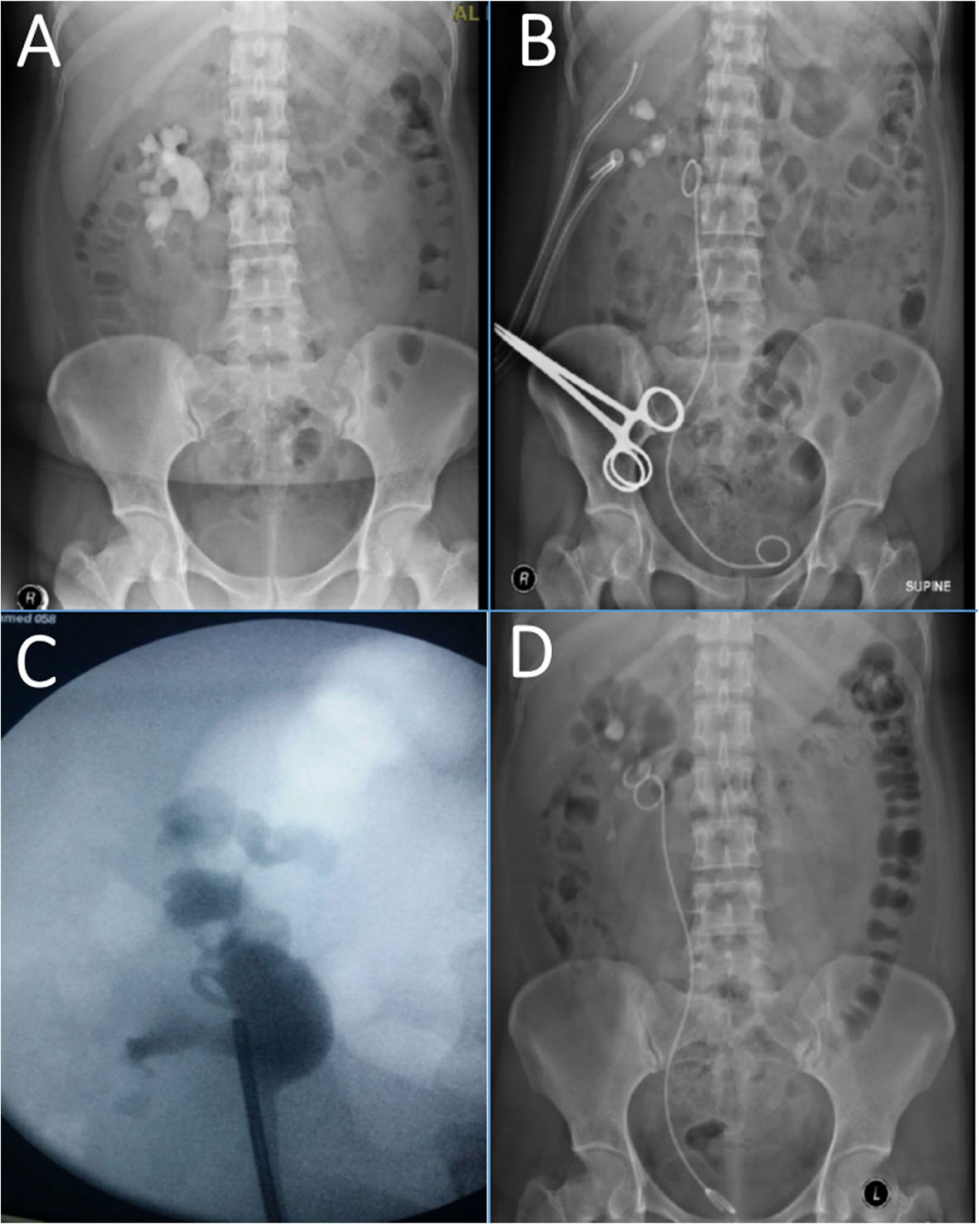
Imaging of the second case. **a**, Pre-operative plain x-ray with full staghorn calculus; **b**, Immediate post-operative plain x-ray showing the two nephrostomy drainage tubes clamped and the JJ stent in position with the remnant stone fragments; **c**, Retrograde pyelography showing the loop of the JJ stent outside the collecting system; **d**, Plain x-ray showing isolated upper coil of the embedded stent following transection of the stent at the junction of the coil with the stem using rigid ureteroscopy. Another JJ stent was placed during the procedure

A 32 years old female presented with right flank pain and a history of urinary tract infections. Imaging revealed a large right staghorn stone (Fig. 2a). So, she underwent prone PNL using Amplatz dilators. Three ports were used and similar to the first patient, there was a perforation of the renal pelvis by the tip of the sheath. The perforation was large and in view of the excessive bleeding and the fear of significant extravasation, the procedure was stopped before total clearance of the stone. The collecting system was then drained by two nephrostomy tubes and antegrade placement of a JJ stent (Fig. 2b). Post-operatively, she did well, and the two drains were removed on the second post-operative day. In view of the remaining fragments, she opted to undergo fURS and laser lithotripsy 6 weeks following the PNL. However, trial to remove the JJ stent failed and the stent was found to be embedded in the renal tissues as shown by the retrograde pyelography (Fig. 2c). Therefore, using a rigid ureteroscope, the stent was transected at the junction of the upper coil with the stem and a trial to perform a fURS failed due the inability to pass an access sheath (12/14Fr). So another stent was placed (Fig. 2d) until 8 weeks later when she was consented for PNL but she asked if she could have one more trial of fURS in the same sitting. By that time, and by using a smaller access sheath (10/12Fr which was not available in the first session), fURS was performed and the JJ stent upper coil was released by laser incision of the tissues trapping the stent coil. The stent coil was removed completely. At the same session, she underwent laser lithotripsy of the remaining stones and the procedure was uneventful with smooth post-operative recovery.

## Discussion and conclusion

Large disruption of the collecting system in PNL might require cessation of the procedure and adequate drainage using different methods including placement of JJ stents [2]. The insertion and use of JJ stents is well known to be associated with several complications such as misplacement, renal parenchymal perforation etc. [4, 5] but burring of the upper coil of the JJ stent in the fibrous tissues at the site of collecting system perforation has not been previously reported.

In the two cases, the JJ stents were inserted and the upper coils were placed in the renal pelvis close to the perforation site. It appears that the embedding of the upper coils of the stents have happened due to the formation of fibrous tissues over a trapped portion of the stent upper coil instead of healing from below and keeping the stent inside the collecting system. In the first case, the fact that the renal pelvis was very narrow to accommodate the upper coil had contributed to this complication and entrapment of the coil in the perforation site. In the second case, the large nature of injury has probably led to the entrapment of the stent coil.

The fibrous tissues entrapping the stent could be hard and difficult to incise. In the first case, it was not possible to incise the tissues over the stent using fURS due to difficult maneuvering and lack of space in the narrow renal pelvis. This required a percutaneous approach to do so. In the second case, although, it was possible to incise the fibrous tissue using fURS and laser, this required two sessions due to very tight ureter and inability to pass an access sheath in the first session.

Although the release of the stents was uneventful and was not associated with bleeding or other complications, this has required extra-procedures. To prevent such complication, we suggest trying to avoid renal system perforation by careful use of the sheath and the nephroscope and minimizing the rotational movement of the scope during PNL. In patients with relatively small stones and narrow renal pelvis such as the first case, mini-PNL would certainly be less traumatic. In cases when the perforation has already occurred, the authors suggest draining the collecting system using nephrostomy tube or percutaneous nephro-ureteral stent. Alternatively, every trial should be made to place the upper coil of the JJ stent away from the perforation site such as placing it in an upper calyx especially if the perforated renal pelvis is relatively small to accommodate the upper coil comfortably.

In conclusion, this is the first presentation of buried JJ stents at the site of previous perforation of collecting system following PNL. To prevent this complication, the authors suggest draining the collecting system using nephrostomy tube. Alternatively, every effort should be taken to place the upper coil of the JJ stent away from the perforation site.

## Data Availability

All imaging are available for review and has been presented.

## Abbreviations

PNL: Percutaneous nephrolithotomy
fURS: Flexible ureterorenoscopy
